# Adjuvant Bidirectional Chemotherapy with Intraperitoneal Pemetrexed Combined with Intravenous Cisplatin for Diffuse Malignant Peritoneal Mesothelioma

**DOI:** 10.1155/2012/890450

**Published:** 2012-08-08

**Authors:** Lana Bijelic, O. Anthony Stuart, Paul Sugarbaker

**Affiliations:** ^1^Department of Surgery, Washington Hospital Center, 110 Irving Street, Washington, DC 20010, USA; ^2^Medstar Health Research Institute, Washington, DC, USA

## Abstract

Cytoreductive surgery (CRS) with heated intraoperative intraperitoneal chemotherapy (HIPEC) has emerged as optimal treatment for diffuse malignant peritoneal mesothelioma (DMPM) showing median survivals of 36–92 months. However, recurrences occur frequently even in patients undergoing optimal cytreduction and are often confined to the abdomen. We initiated a Phase II study of adjuvant intraperitoneal pemetrexed combined with intravenous cisplatin for patients undergoing CRS and HIPEC for DMPM. The treatment consisted of pemetrexed 500 mg/m^2^ intraperitoneally and cisplatin 50 mg/m^2^ intravenously given simultaneously on day 1 of every 21 day cycle for 6 cycles. The primary endpoint of the study was treatment related toxicity. From July 2007 until July 2009 ten patients were enrolled. Nine of 10 completed all 6 cycles of adjuvant treatment per protocol. The most common toxicities were fatigue, nausea and abdominal pain grade 1 or 2. There was one grade 3 toxicity consisting of a catheter infection. The median survival for all 10 patients was 33.5 months. Pharmacokinetic analysis of intraperitoneal pemetrexed showed a peritoneal to plasma area under the curve ratio of 70. Our study shows that adjuvant intravenous cisplatin and intraperitoneal pemetrexed can be used following CRS and HIPEC for DMPM with low morbidity.

## 1. Introduction

Malignant mesotheliomas are tumors arising from the lining of the pleural or peritoneal cavity. Of the estimated 2,500 cases of mesothelioma occurring in the United States annually, approximately 20% are peritoneal mesotheliomas. Peritoneal mesotheliomas are characterized by numerous tumor nodules covering the parietal and visceral serosal surfaces of the peritoneal cavity. Clinically, it usually presents with abdominal distention, ascites, and pain [1]. The natural history of peritoneal mesothelioma is that of rapid progression with fatal outcome without treatment. Mesothelioma remains confined to the serosal surface of the abdominal cavity in the majority of the patients [2, 3]. Treatment approaches have traditionally been largely unsuccessful in this disease and consisted of systemic chemotherapy with surgery employed for palliation of gastrointestinal symptoms. The median survivals with these strategies generally ranged from 9 to 14 months [4–6]. 

An alternative treatment strategy consisting of more aggressive surgery and local-regional chemotherapy aimed at complete eradication of the disease has emerged showing dramatically improved median survivals. Cytoreductive surgery (CRS) with heated intraoperative intraperitoneal chemotherapy (HIPEC) has been used in a number of centers worldwide showing median survivals of 36–92 months and has become the preferred therapy for eligible patients [7–12]. Yet, a significant proportion of patients with mesothelioma treated with this modality are not able to achieve complete cytoreduction and therefore need further treatment with chemotherapy. Even in patients who have a complete cytoreduction and HIPEC, recurrent disease is common and it usually occurs in the abdomen [13]. Pemetrexed is a multi-targeted antifolate agent that was shown in Phase III studies to significantly improve response rates in patients with advanced pleural and peritoneal malignant mesothelioma. Treatment was with systemic cisplatin combined with pemetrexed compared with systemic cisplatin alone [14, 15]. In this current study we prospectively assess the feasibility and toxicity of an adjuvant treatment with intraperitoneal pemetrexed combined with intravenous cisplatin in patients with malignant peritoneal mesothelioma who underwent CRS and HIPEC.

## 2. Methods

### 2.1. Patients

Patients with histologically proven diffuse malignant peritoneal mesothelioma who were candidates for CRS and HIPEC at our institution were offered participation in this Phase II study. Eligibility criteria included completion of the best possible surgical cytoreduction, performance status of 0–2, and adequate organ and marrow function. The study was approved by the institutional IRB and all patients signed an informed consent. 

### 2.2. Treatment

This was a single-institution Phase II study of adjuvant intraperitoneal pemetrexed combined with intravenous cisplatin. The primary endpoint of the study was toxicity related to the adjuvant treatment. Secondary endpoint was survival at 2 years.

The treatment consisted of pemetrexed 500 mg/m^2^ given intraperitoneally and cisplatin 50 mg/m^2^ given intravenously simultaneously on day 1 of every 21-day cycle for 6 cycles. Pemetrexed was mixed in 1liter of peritoneal dialysis solution and administered through an implantable peritoneal port (Port-a-Cath, Smith Medical ASD Inc., St. Paul, MN, USA) placed at the time of cytoreductive surgery. 

All patients received folic acid 1000 micrograms orally daily and vitamin B12 1000 micrograms intramuscularly every 9 weeks beginning 2 weeks before starting therapy and continued through the end of the last cycle of therapy. The patients also received dexamethasone orally on the day before, the day of, and the day after pemetrexed.

### 2.3. Peritoneal Port Placement and Maintenance

Peritoneal ports were placed at the time of CRS and HIPEC in all patients. Immediately prior to abdominal closure, the port was placed using the following technique: a 5 cm transverse incision was made lateral to the umbilicus on the left side overlying the lateral border of the rectus sheath. The tissues were dissected to the abdominal fascia and a small opening made in the fascia to accommodate the catheter that was placed in the abdomen with the tip directed at the pelvis. Blunt dissection was used to create a subcutaneous channel and pocket 10 cm cephalad to the skin incision where the port was positioned. A right angle noncoring needle (Gripper Plus, Smith Medical ASD Inc., St. Paul, MN, USA) was then used to access the port, secured, in position with sutures and left in place for 10 days during postoperative recovery to prevent port twisting.

### 2.4. Clinical Data Collection and Statistical Analysis

Details regarding the extent of peritoneal involvement by mesothelioma, completeness of cytoreduction, and HIPEC treatment received were recorded prospectively in the HIPEC database for all patients. Toxicities related to adjuvant treatment were prospectively recorded using the Common Toxicity Criteria for Adverse Events Version 3. Survival was defined as the time from cytoreductive surgery to the time of death from any cause. Patients who were alive at the time of last followup were censored on that date. Statistical analysis was performed using SPSS software.

## 3. High-Performance Liquid Chromatography (HPLC) Analysis of Plasma, Peritoneal Fluid, and Urine

### 3.1. Sampling

Prior to treatment a 3 mL reference sample of the chemotherapy solution was obtained along with a 3 mL sample of blood and urine. Subsequently, 3 mL aliquots of blood, peritoneal fluid, and urine were obtained at 15-minute intervals for one-hour and 30-minute intervals for an additional two hours in all patients. These samples were centrifuged to remove debris or red blood cells. The cell-free solutions were frozen and stored for high performance liquid chromatography (HPLC) analysis which was performed within one week.

Pemetrexed concentrations were determined using a modified version of the HPLC method as described by Neurnberg et al. [16]. Briefly, the HPLC system consisted of a Shimadzu LC7A instrument equipped with an SPD-6AV (UV-VIS) detector set at 295 nm and a C-R6a “Chromatopac” data processor (Shimadzu Instruments, Columbia, MD, USA). Chromatographic separation was accomplished on a C18 reversed phase column (Varian Associates, Walnut Creek, CA, USA). The mobile phase consisted of 28% acetonitrile in 0.1% orthophosphoric acid with 0.1% triethylamine. The flow rate was 1.2 mL/min and the sample injection volume was 50 *μ*L.

All samples were thawed at room temperature before HPLC analysis. Peritoneal fluid samples were diluted appropriately with methanol. After thorough mixing the resulting solutions were filtered through 0.45 micron syringe filters prior to HPLC injection.

For plasma samples, a 500 *μ*L sample was mixed with 10 volumes of chloroform-isopropanol (2 : 1) in 15 mL screw-capped polypropylene centrifuge tubes. After thorough mixing followed by centrifugation the lower organic phase was transferred to a clean polypropylene centrifuge tube and evaporated to dryness under a stream of N2 at 37°C. The residue was dissolved in 250 *μ*L of methanol and filtered through a 0.45 micron syringe filter prior to HPLC injection.

### 3.2. Data Retrieval and Statistics

All data presented on the graphs are mean +1 standard deviation. Calculations of area under the curve (AUC) and subsequent AUC ratios were obtained using GraphPad Prism analyses (GraphPad Software, Inc., La Jolla, CA, USA). 

## 4. Results

### 4.1. Demographic Features and Outcome of Cytoreductive Surgery

From July 2007 until July 2009, ten patients signed the informed consent and were enrolled in the Phase I/II study. All patients had histologically confirmed diffuse malignant peritoneal mesothelioma: 8/10 had epithelioid type while 2 had biphasic histology. All ten patients underwent CRS by the same surgeon and received HIPEC consisting of 50 mg/m^2^ of cisplatin combined with 15 mg/m^2^ of doxorubicin in 1.5 L/m^2^ of peritoneal dialysis solution circulated for 90 minutes at 41–42.5°C. Nine of the 10 patients also received early postoperative intraperitoneal chemotherapy (EPIC) consisting of paclitaxel 20 mg/m^2^ daily for 5 days starting on postoperative day 1.  Table 1 summarizes the baseline characteristics of the patients and outcomes of CRS.

### 4.2. Bidirectional Adjuvant Chemotherapy

Nine of 10 patients were able to complete all 6 cycles of therapy without treatment delays or dosing modifications. One patient developed a catheter infection after cycle number 3 and required catheter removal. He was switched to intravenous pemetrexed and cisplatin for one cycle, then had a new peritoneal catheter placed and subsequently completed cycles 5 and 6 according to protocol. The most common observed toxicities were fatigue, nausea, and abdominal pain but were generally mild. The only Grade 3 toxicity was the above mentioned catheter infection. There were no deaths related to treatment and no hospitalizations due to treatment side effects.  Table 2 summarizes the toxicities observed in the 10 patients enrolled.

### 4.3. Pharmacokinetics of Intraperitoneal Pemetrexed

**Figure 1:**
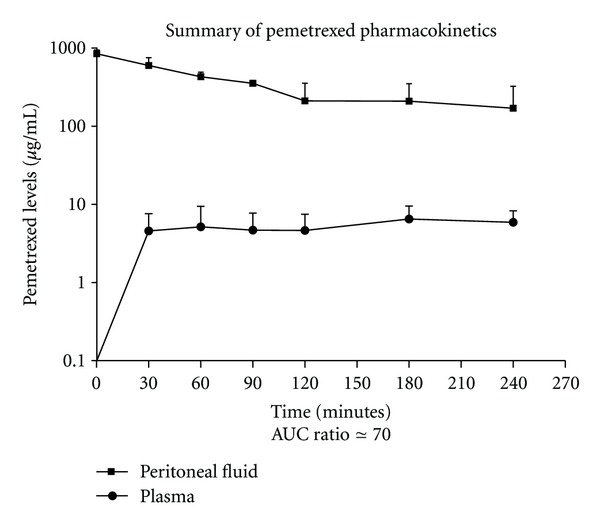
Concentration times time graph of pemetrexed in peritoneal fluid and plasma from four different pharmacologic studies. The AUC ratio of peritoneal fluid to plasma was 70. Peak plasma concentration was 0.05 (+0.02) *μ*g/mL at 30 minutes.

In four patients the pharmacokinetics of intraperitoneal pemetrexed was studied on the first cycle of adjuvant treatment (Figure 1). The area under the curve of peritoneal fluid concentration times time was 84150 *μ*gmL^−1^. The area under the curve of plasma pemetrexed concentrations times time was 1250 *μ*gmL^−1^. The increased exposure of peritoneal surfaces to chemotherapy as compared to plasma (area under the curve ratio) was 70. The peak plasma concentration was 6.5 ± 3 *μ*g/mL at 180 minutes.

**Figure 2:**
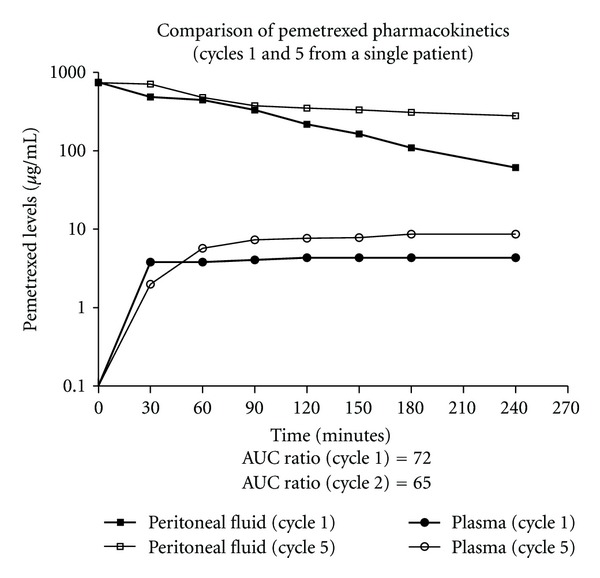
Concentration times time graph of intraperitoneal pemetrexed in peritoneal fluid and plasma in a single patient who had pharmacologic studies of the first and fifth cycle.

In a single patient the first and final treatments with intraperitoneal pemetrexed were studied pharmacologically. The data is shown in Figure 2.

### 4.4. Follow-Up Data

After a median followup of 44 months, 4 patients have no evidence of disease, 2 are alive with disease, and 4 have died of disease. The median survival for all 10 patients is 33.5 months. With a median followup of 50 months in 6 living patients, no long term symptoms of peritoneal sclerosis have been observed.

## 5. Discussion

DMPM is a rare malignancy of the abdominal cavity characterized by extensive involvement of the peritoneal surfaces by tumor nodules. Due to the diffuse nature of the tumor, it has traditionally been considered not appropriate for surgical intervention and treated with palliative measures [17, 18]. The development of the peritonectomy procedures combined with HIPEC allowed many patients with malignant peritoneal mesothelioma to undergo a potentially curative treatment [18]. The results of cytoreductive surgery combined with HIPEC have been reported by several international centers showing significantly longer median survivals compared to historical results of treatment using palliative systemic chemotherapy with or without abdominal radiation [7–12, 18, 19]. Recently, the results of a multi-institutional registry of patients with DMPM treated with CRS and HIPEC at eight leading institutions over the last 20 years was published. Four hundred five patients were included and 46% were able to have a complete or near-complete cytoreduction [20]. The median survival was 53 months and 5-year survival was 47%. The prognostic factors associated with survival were epithelial histologic subtype, absence of lymph node metastasis, completeness of cytoreductive score, and HIPEC [17].

While this large registry data provides encouraging results overall, it also clearly shows that CRS and HIPEC are unlikely to be sufficient therapy in the majority of patients as only 46% were able to have a complete cytoreduction. Other experienced institutions have shown that even in the group of patients who receive a complete cytoreduction, local regional recurrence is common. Baratti et al. were able to achieve a complete or near-complete cytoreduction in 56 of 70 patients but despite that 38 developed recurrent disease including 11of 26 patients in the CC-0 group. Importantly, treatment failures were primarily confined to the abdominal cavity in the vast majority of patients [13].

Adjuvant treatment following CRS and HIPEC for patients with DMPM appears to be needed for the patients with high grade disease but there is no consensus regarding the optimal approach. Large randomized trials of palliative systemic therapy in mesothelioma have shown that combination therapy using cisplatin and pemetrexed has superior response rates and median survival compared to cisplatin alone [14]. Therefore, we designed our study to assess the feasibility of an adjuvant treatment plan using the current standard agents but administered regionally in order to achieve maximum benefit at the sites most at risk while minimizing systemic toxicity. In ongoing attempts to improve the local-regional control of peritoneal mesothelioma after CRS and HIPEC, we elected to explore in a Phase I/II study the intraperitoneal used of pemetrexed.

Pemetrexed is a multitargeted antifolate that inhibits dihydrofolate reductase, thymidylate synthase, and glycinamide ribonucleotide formyltransferase, key enzymes involved in purine and pyrimidine synthesis [21, 22]. With a molecular weight of 471.384, it is a drug expected to have a favorable profile for intraperitoneal administration based on the principles described by Dedrick et al. [23]. We have previously studied the pharmacokinetics of pemetrexed after intraperitoneal administration in a rat model showing a 24-fold increase in exposure of peritoneal surfaces to pemetrexed compared to intravenous administration [24]. Therefore, there is a strong pharmacologic and clinical rationale for choosing the intraperitoneal route for novel approaches to adjuvant treatment of peritoneal mesothelioma following CRS and HIPEC. 

In this study, we confirmed that the exposure of peritoneal surfaces to adjuvant pemetrexed was 70 times greater than plasma exposure. This suggests a role for continued local-regional adjuvant treatment of peritoneal mesothelioma patients judged to be a high risk for recurrence after CRS and HIPEC. Peritoneal mesothelioma, a disease largely confined to the abdominal and pelvic space throughout its natural history is not the only disease to be treated by adjuvant bidirectional chemotherapy.

The intraperitoneal route for adjuvant chemotherapy in cancers with a high propensity for progression on peritoneal surfaces has been most extensively studied in ovarian cancer. The large Gynecologic Oncology Group 172 randomized trial showed a significant benefit in overall survival and progression-free survival for patients with optimally debulked stage III epithelial ovarian cancer treated with intraperitoneal and intravenous chemotherapy compared to patients treated with intravenous chemotherapy alone [25]. 

This has led to a more widespread use of intraperitoneal chemotherapy in the adjuvant setting but there is still significant resistance to fully integrate this route of administration into daily practice. In great part, this is due to concerns about potential complications and significant morbidity related to intraperitoneal chemotherapy administration and the intraperitoneal catheter. Catheter-associated complications were the primary cause for failure to complete all six cycles of therapy in the GOG 172 trial: 119 (58%) of patients did not complete all six cycles of chemotherapy in the study and of these and 40 (34%) failed because of catheter complications [26]. Rectosigmoid colon resection was associated with failure to initiate intraperitoneal chemotherapy. In our study, only 1 significant catheter-related problem was observed: this was a catheter infection that required removal and a change to intravenous chemotherapy for one cycle. This patient also had a rectosigmoid colon resection as part of the cytoreductive surgery. It is possible that an increased risk of catheter infection is seen in patients who undergo rectosigmoid colon resection at the time of cytoreductive surgery because of possible contamination. Special attention should be given to the placement of the port in those circumstances. 

The intraperitoneal administration of chemotherapy at the same dose used for systemic administration has been reported to be associated with few systemic side effects, due to differences in the pharmacokinetics [27]. This study is in accordance with this principle showing that few significant systemic toxicities were observed. Local-regional toxicities related to abdominal distention and discomfort at the time of administration were also low in our study. This is probably due to the fact that we kept the volume of the intraperitoneal chemotherapy solution at a moderate amount of 1 liter, which is well tolerated by most patients.

A single patient was studied pharmacologically on her first and sixth cycles of intraperitoneal pemetrexed. Maintenance of the pharmacologic advantage throughout the treatment was suggested by similar area under the curve ratios of both studies. Also, long-term followup of our patients up to four years does not suggest an intestinal fibrosis resulting from these intraperitoneal chemotherapy treatments.

At the initiation of this Phase I/II study, a dose escalation of intraperitoneal pemetrexed was planned. However, as the patient went on to complete all six cycles of treatment the fatigue they experienced did not suggest that a dose escalation was possible. A larger dose of chemotherapy, in our judgment, would have seriously jeopardized the completion of the protocol treatments. The regimen, as completed in these protocol patients, has now become the standard of care at our institution.

In summary, our study shows that an adjuvant protocol of combined intravenous and intraperitoneal chemotherapy can be successfully implemented for patients with peritoneal mesothelioma following CRS and HIPEC with low morbidity. Our practice of placing the intraperitoneal port at the end cytoreductive surgery was successful and only one significant catheter problem was observed. We recommend our regimen, tested as a multi-institutional adjuvant intraperitoneal pemetrexed combined with intravenous cisplatin adjuvant therapy, for patients with this rare cancer.

## Figures and Tables

**Table 1:** Demographic features and outcomes of cytoreductive surgery in ten patients undergoing adjuvant chemotherapy with intraperitoneal pemetrexed and intravenous cisplatin for diffuse malignant peritoneal mesothelioma.

	Number of patients
Total	10
Female	3
Male	7
Age
Mean	51
Range	23–69
Peritoneal cancer index (PCI)
Mean	24
Range	7–39
CC score
CC 0/1	4
CC 2	4
CC 3	2
Visceral sparing cytoreduction	8
Colon resection	2

**Table 2:** Toxicities observed in ten patients treated with adjuvant intraperitoneal pemetrexed combined with intravenous cisplatin following cytoreductive surgery and hyperthermic intraperitoneal chemotherapy for diffuse malignant peritoneal mesothelioma.

	Grade I-II	Grade III-IV
Nausea	5	0
Abdominal pain	5	0
Alopecia	2	0
Fatigue	6	0
Neutropenia	0	0
Thrombocytopenia	0	0
Catheter infection	0	1
